# Sailuotong Capsule Prevents the Cerebral Ischaemia-Induced Neuroinflammation and Impairment of Recognition Memory through Inhibition of LCN2 Expression

**DOI:** 10.1155/2019/8416105

**Published:** 2019-09-03

**Authors:** Yehao Zhang, Jianxun Liu, Mingjiang Yao, WenTing Song, Yongqiu Zheng, Li Xu, Mingqian Sun, Bin Yang, Alan Bensoussan, Dennis Chang, Hao Li

**Affiliations:** ^1^Institute of Basic Medical Sciences of Xiyuan Hospital, China Academy of Chinese Medical Sciences, Beijing Key Laboratory of Pharmacology of Chinese Materia, Beijing 100091, China; ^2^NICM, Western Sydney University, Penrith, NSW 2751, Australia

## Abstract

**Background:**

Astrogliosis can result in astrocytes with hypertrophic morphology after injury, indicated by extended processes and swollen cell bodies. Lipocalin-2 (LCN2), a secreted glycoprotein belonging to the lipocalin superfamily, has been reported to play a detrimental role in ischaemic brains and neurodegenerative diseases. Sailuotong (SLT) capsule is a standardized three-herb preparation composed of ginseng, ginkgo, and saffron for the treatment of vascular dementia. Although recent clinical trials have demonstrated the beneficial effect of SLT on vascular dementia, its potential cellular mechanism has not been fully explored.

**Methods:**

Male adult Sprague-Dawley (SD) rats were subjected to microsphere-embolized cerebral ischaemia. Immunostaining and Western blotting were performed to assess astrocytic reaction. Human astrocytes exposed to oxygen-glucose deprivation (OGD) were used to elucidate the effects of SLT-induced inflammation and astrocytic reaction.

**Results:**

A memory recovery effect was found to be associated with the cerebral ischaemia-induced expression of inflammatory proteins and the suppression of LCN2 expression in the brain. Additionally, SLT reduced the astrocytic reaction, LCN2 expression, and the phosphorylation of STAT3 and JAK2. For *in vitro* experiments, OGD-induced expression of inflammation and LCN2 was also decreased in human astrocyte by the SLT treatment. Moreover, LCN2 overexpression significantly enhanced the above effects. SLT downregulated these effects that were enhanced by LCN2 overexpression.

**Conclusions:**

SLT mediates neuroinflammation, thereby protecting against ischaemic brain injury by inhibiting astrogliosis and suppressing neuroinflammation via the LCN2-JAK2/STAT3 pathway, providing a new idea for the treatment strategy of ischaemic stroke.

## 1. Introduction

The incidence of stroke around the world has reached epidemic levels. In the past two decades, there have been marked increases in strokes, the number of living stroke victims, the population-level loss of life (called disability-adjusted life years or DALYs), and the number of deaths related to strokes [1]. Lasting physical debility and cognitive deterioration are experienced by most stroke survivors [2]. Stroke patients, who endure an acute period of infarction, must then cope with ongoing neuroinflammation and the associated neurological impairment. Inflammation is a serious aspect of the disease development of ischaemic stroke and other types of ischaemic brain injury. These injuries are caused by a decrease in blood circulation, followed by the activation of intravascular leukocytes and the concomitant release of inflammatory cytokines from the ischaemic brain parenchyma and endothelium, all of which have the potential to increase the damage to central nervous system (CNS) tissues [3].

The CNS contains many resident cells that originate from nerve epithelial cells, collectively known as neuroglia. Neuroglial cell subtypes include astrocytes, oligodendrocytes, and polyglia [4]. Astrocytes actively maintain the immune response following CNS ischaemia through the production of complement components, inflammatory mediators such as interleukin- (IL-) 6 and IL-1*β*, and chemokines, including C-X-C motif chemokine ligand 12 (CXCL12), CXCL1, CXCL10, monocyte chemoattractant protein (MCP)-1, and lipocalin-2 (LCN2) [5, 6]. After prolonged activation, reactive astrocytosis (also called astrogliosis) results in a structure called a glial scar, which is the demarcation line between an ischaemic core and the healthy surrounding brain tissue [7]. Activated astrocytes release the glycoprotein LCN2, which mediates certain biological processes, including cell death [8], cell migration [9], and innate immunity [10]. Recent studies have indicated that brain injury or infection can result in neuroinflammation that may encourage the release of LCN2 from astrocytes [11, 12]. Similarly, studies have also shown that continuing and disproportionate immune responses can exacerbate the secretion of inflammatory cytokines, such as LCN2, with a subsequent aggravation of neural inequity in the hippocampus, leading to long-term behavioural impairments [13, 14]. Therefore, targeting astrocytic LCN2 may be a prospective molecule-level therapy for clinicians treating inflammatory CNS impairments.

The use of herbal medicine to treat cerebrovascular diseases in Asia has persisted for centuries. The Sailuotong (SLT) capsule is a standardized herb preparation composed of *Panax ginseng* (ginseng), *Ginkgo biloba* (ginkgo), and *Crocus sativus* (saffron) in three specific doses that is used to treat vascular dementia (VaD) [15]. Each of these herbal components have been proven to avert and/or treat circulatory diseases such as hypertension and stroke. For example, a standardized extract of *G. biloba* (EGB 761) has antioxidant and antiplatelet characteristics and can reduce cerebral ischaemic injury [16–20]. Other studies have shown that the ginsenosides Rg1, Rb1, and Rg2 have neuroprotective effects [21–24].

Although the most recent clinical study showed that SLT improves cognition and surveillance in patients [25], and many preclinical studies have demonstrated a cerebrovascular protective effect of its single components, the mechanism by which SLT regulates astrocyte activities through its anti-inflammatory effects has not been previously studied. Here, we investigated two questions: does SLT treatment alleviate reactive astrocytes and, thus, mediate neuroinflammation, and if so, does the benefit of SLT treatment involve the inhibition of Janus kinase-2 (JAK2) signal transducer and activator of transcription-3- (STAT3-) mediated LCN2 expression?

## 2. Materials and Methods

### 2.1. Rat Model of Microsphere-Induced Cerebral Embolism

Adult male Sprague-Dawley rats (weighing 220–250 g) were used in this study (Beijing Vital River Laboratory Animal Technology Co. Ltd., Beijing, China). The animal protocols were carried out according to the recommendations of the Chinese Academy of Medical Sciences Committee for Experimental Animal Use and Care. The procedures used were designed to reduce or diminish the quantity and discomfort of the animals tested. All rats were kept on a 12-hour light/dark cycle in standard conditions (23 ± 1°C and 55 ± 5% humidity) with access to water and food *ad libitum*.

Forty-six adult SD rats were arbitrarily separated into four groups. Cerebral embolism was induced using the microsphere method as previously described [20]. In short, the rats were anaesthetized with chloral hydrate (40 mg/kg), and the common carotid artery, internal carotid artery, and right external carotid were separated using forceps. The common carotid artery was clipped with an artery clamp, and the distal end of the external carotid artery was ligated with a thread. Fluorescence microspheres (106–212 *μ*m in diameter, UVPMS-BY2, Cospheric, USA) were injected into the external carotid artery with a syringe, and the artery clamp was simultaneously released, allowing the microspheres to travel to the various arteries of the brain and cause embolisms. The proximal end of the external carotid artery was then ligated with a thread, and the wound was sutured layer by layer. In the control group, rats received the same amount of serum without microspheres.

### 2.2. Drug Administration

SLT was supplied by the Shineway Pharmaceutical Group (Shijiazhuang, China). Extracts of ginseng (20170301, Panax ginseng C.A. Meyer), ginkgo (20170122, Ginkgo biloba L.), and saffron (20170320, Crocus sativus L.) were prepared in a Good Manufacturing Practice-certified facility (Shineway Pharmaceutical Group, Hebei, China). Ultraviolet (UV) spectroscopy or HPLC-UV was used to measure the composition of ingredients to regulate the quality of the ingredients. The readings showed that 77% of the total ginsenosides were found in the ginseng extract (UV), with ginsenoside Rg1, Re, and Rb1 present at 5.5%, 3.2%, and 13.1%, respectively (HPLC-UV); 49% of the total flavones were found in the extract (UV); 28.7% of the total glycosides were found in the extract, including aglycone of quercetin, isorhamnetin, and kaempferol (HPLC-UV); 11.6% of the total ginkgolides A, B, and C, and bilobalide, were found in the ginkgo extract, with 3.3% of ginkgolide A (HPLC-UV) among them; and 65% of the total crocins were found in the saffron extract (UV), including 27% of crocin-1 (HPLC-UV) [26]. SLT was prepared from the above three extracts to a specific formulation and was then administered intragastrically at doses of 16.5 and 33 mg/kg for 28 days. Each rat in the control group and the model group was given an identical volume of saline intragastrically every day.

### 2.3. Evaluation of Neurological Deficits

Neurological deficit scores were calculated 2 hours and 24 hours after ischaemia in each group using a previously published five-point system [27]. Specifically, a rat that exhibited normal spontaneous movement (without obvious neurological defects) received a 0, a rat that could not fully extend its right paw received a 1, a rat that circled clockwise received a 2, a rat that fell to the right received a 3, and a rat that could not walk received a 4. The deficit scores were assigned by an investigator who was unaware of the groups.

### 2.4. Morris Water Maze (MWM) Test

The MWM was used to evaluate three-dimensional spatial memory and learning in all four groups of rats [28]. The water maze was a circular pool (330 cm in diameter and 60 cm high containing water at a depth of 45 cm at 22°C ± 1°C). A nontoxic black ink was introduced to make the water opaque. The pool was divided into equal quadrants, each containing four points (north, east, south, and west). A round platform (10 cm in diameter) was painted black and hidden by being submerged 1.5 cm in the southwest quadrant. The location of the training platform remained unchanged during the experiment. The swimming paths of the rats were recorded with digital imaging equipment.

### 2.5. Measurement of Cerebral Infarction

For the evaluation of the success of the cerebral ischaemia model, the rats were anaesthetized with chloral hydrate (40 mg/kg) twenty-four hours after surgery (*n* = 3 in the control and test groups). Blood was taken from the abdominal aorta before decapitation and the quick removal of the brain. The brains were stored in a cold, oxygenated physiological salt solution. Coronal slices (1 mm) were attained and fixed for 10 min in prewarmed 2% triphenyltetrazolium chloride (TTC). The slices were then fixed for 30 min in 10% paraformaldehyde.

### 2.6. Culture and Transfection of Human Astrocytes

Human astrocytes (ScienCell Research Laboratories, CA, USA) were cultured in astrocyte media (AM) (ScienCell, USA) in a humidified, 5% CO_2_ atmosphere at 37°C. After cells reached 70% confluence, the astrocytes were stably transfected using the LCN2 gene expression vector (EX-m0282-Lv201), the control expression vector (EX-NEG-Lv201), and 7.5 *μ*l/ml of the lentiviral vector (LPP-m0282-Lv201-100) (GeneCopoeia, Rockville, USA) for 24 hours. Following transfection, the cells were kept in fresh AM without the lentiviral vector for 48 hours.

### 2.7. Oxygen-Glucose Deprivation (OGD) Management and Treatment

OGD experiments were performed as based on a previous method [20]. After purification and transfection, the cells were placed in a premixed gas (94% N_2_, 5% CO_2_, and 1% O_2_) culture box and cultured in deoxygenated DMEM without glucose and fetal bovine serum (FBS) for 6 hours. After 6 hours, normal AM with 10% FBS serum was given, and cells were transferred to an atmosphere incubator for 12 hours. The control group cells were cultured with normal AM. During OGD stimulation, cells were treated for 6 hours with 2.5, 5, or 10 mg/l SLT.

### 2.8. Western Blotting

Protein was extracted from the ischaemic penumbra with RIPA buffer (Beyotime Biotechnology, Shanghai, China) 28 days after cerebral ischaemia and combined with a protease and phosphatase inhibitor cocktail (MCE, New Jersey, USA). The tissue was cut into fine fragments, completely homogenized with a sonifier, and then centrifuged (10000-14000 g, 3-5 min), and the supernatant was collected for subsequent experiments. Protein of equivalent molecular weight was loaded into SDS-PAGE gel wells and transferred from the gel to polyvinylidene difluoride (PVDF) membranes. Nonspecific sites were blocked for 60 min with 5% bovine serum albumin (BSA) in TBST buffer, and the blots were then incubated overnight at 4°C with antibodies against LCN2 (Abcam, 1 : 2000 dilution), phosphorylated- (p-) JAK2 (Tyr1007/1008) (Abcam, 1 : 1000), glial fibrillary acidic protein (GFAP; Proteintech, 1 : 2000), JAK2 (Abcam, 1 : 2000), p-STAT3 (Tyr705) (Cell Signaling Technology, 1 : 2000), STAT3 (Cell Signaling Technology, 1 : 2000), and *β*-actin (Sigma, 1 : 5000) in TBST. Antibody binding was detected with anti-rabbit horseradish peroxidase- (HRP-) conjugated immunoglobulin G (IgG; 1 : 1000) in TBST (60 min, room temperature). The reaction bands were detected using the ECL detection reagent per the manufacturer's instructions (Thermo Fisher Scientific, MA, USA). The extraction procedure for cell protein was the same as the above steps.

### 2.9. Immunofluorescence Analysis and Hematoxylin-Eosin (HE) Staining

The brains were dissected and fixed in paraffin. Coronal cryostat sections (20 *μ*m) were stained with HE and immunofluorescent dyes. Brain sections were fixed at 4°C for 24 hours in 4% paraformaldehyde in PBS (0.01 M, pH 7.4), dehydrated in a series of graded alcohol dehydrations, and fixed in paraffin. The tissues were sectioned (5 *μ*m) with a Leica® RM1850 rotary microtome (Leica Microsystems, Germany). The paraffin sections were dried at 60°C, dewaxed, and subjected to antigen retrieval. The sections were exposed for 1 hour at 37°C to primary antibodies targeting the following proteins: LCN2 (Abcam, 1 : 200), GFAP (Proteintech, 1 : 200), p-JAK2 (Tyr1007/1008) (Abcam, 1 : 100), and p-STAT3 (Tyr705) (Cell Signaling Technology, 1 : 200). The sections were washed twice with ice-cold PBS and then saturated with fluorescent secondary antibodies (Cell Signaling Technology, 1 : 100) in the dark for 1 hour. DAPI (4′,6-diamidino-2-phenylindole) (1 : 1000) was added in the dark for 2 min. The DAPI was rinsed 3 times with PBS for 1 min. The staining procedure for the cells was the same as above. For HE staining, sections were stained with alum HE. Colour images were obtained using a 20x laser scanning confocal microscope (Olympus FV1200, Tokyo, Japan).

### 2.10. Determination of Chemokine/Cytokine Expression in Brain Extracts, Serum Samples, and Astrocytes

A MILLIPLEX® MAP Rat Cytokine/Chemokine Magnetic Bead kit (Millipore, USA) was used per the manufacturer's instructions to quantify the concentrations of chemokines and cytokines in the cerebral hemisphere extracts, serum samples, and astrocytes. The protein cerebral hemisphere of concentration was quantitated by the BCA protein assay (Invitrogen). The protein concentration was quantified to 16 mg/ml. The chemokines and cytokines in ischaemic penumbra tissues were collected 28 days after cerebral ischaemia in EMD Millipore's buffer. Blood serum was collected from the abdominal aorta. The chemokines and cytokines from astrocytes that had been conditioned in medium were collected after OGD induction. The following cytokines were analysed: tumor necrosis factor-*α* (TNF-*α*), IL-1*α*, IL-1*β*, IL-12, and IL-6 and the chemokine CXCL10 (IP-10). A FLEXMAP 3D™ system was used to determine the median fluorescence intensity. The cytokine and chemokine levels in brain homogenates, serum samples, and cell samples were subjected to five-parameter logistic analysis.

### 2.11. Statistical Analyses

Data management and analysis were performed using GraphPad Prism software (San Diego, CA). All data are reported as the means ± SD. Each experiment was repeated three times. Univariate analysis of variance (ANOVA) was used for multiple comparisons. Student's *t*-test was conducted to analyse intergroup comparisons. Two-way ANOVA was used in the neurologic study and MWM test to compare the time functions between groups. *P* values of <0.05 were reported as statistically significant.

## 3. Results

### 3.1. Protection from Cerebral Ischaemia-Induced Brain Injury

**Figure 1:**
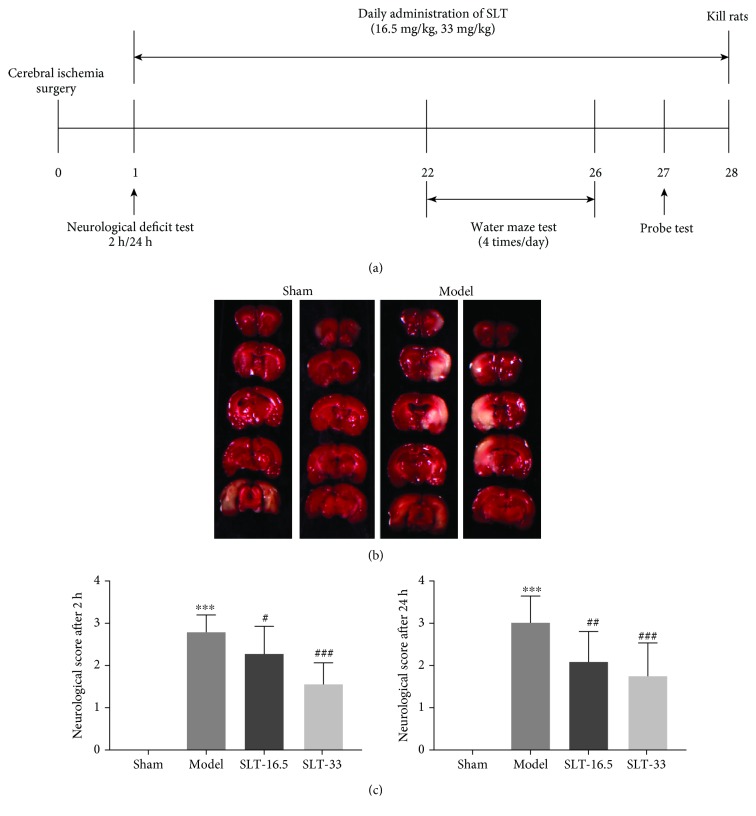
SLT treatment reduced ischaemic infarct volume in the cerebral ischaemia model. (a) Timeline depicts the treatment of SLT and assessments of the cognitive functions of rats. The male rats (*n* = 10) were orally treated with SLT at a daily dose of 16.5 mg/kg and 33 mg/kg for 4 weeks. After surgery for 22 days, memory tests were conducted. The training trial was performed four times a day for 5 days. (b) Cerebral infarct volume was assessed via TTC staining 24 h after cerebral ischaemia. Neurological score (c) of rats after cerebral ischaemia was assessed using a five-point scale system. Data are expressed as the mean ± SEM (*n* = 10). ^∗∗∗^*P* < 0.001 vs. sham group; ^#^*P* < 0.05, ^##^*P* < 0.01, and ^###^*P* < 0.001 vs. the model groups.

SLT was intragastrically administered for 28 days. Cerebral infarction volume was measured by the TTC method 24 hours after surgery. The resulting neurological deficit scores were indicative of protective capabilities of SLT. In comparison to the cerebral ischaemia group, the SLT-16.5 (16.5 mg/kg, daily) and SLT-33 (33 mg/kg, daily) groups showed significantly lower scores (*P* < 0.05). Additionally, the score in the SLT-33 group was less than that in the SLT-16.5 group. The scores for all groups at 2 and 24 hours after surgery and the incidence of cerebral ischaemia are shown in Figure 1(c). The surgery model functioned as expected, as rats with induced cerebral ischaemia displayed obvious cerebral infarctions, whereas rats in the control group showed no signs of cerebral injury.

Figures 1(a) and 1(b) show the infarct volume of the control and test groups at 24 hours after cerebral ischaemia. The striatum, hippocampus, and cortex showed extensive lesions in the model group, whereas those brain regions in the SLT group (33 mg/kg) showed significant differences from those in the model group, in agreement with the neurological deficit scores.

### 3.2. SLT Reduces Brain Damage after Surgery

**Figure 2:**
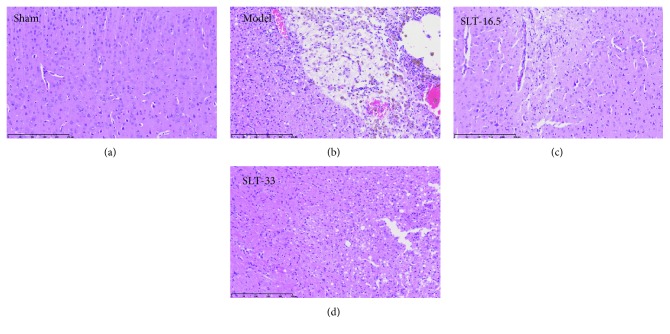
The ischaemic penumbra area in the box was assessed for neuronal apoptosis using HE staining. Cortex sections and hippocampal regions stained with HE presented with neuronal loss and signs of cerebral edema, and swollen cells were observed in the ipsilateral hippocampus; plentiful apoptotic neurons were observed with karyopyknosis, cell gaps, and debris. SLT (33 mg/kg, daily) significantly alleviated the symptoms of apoptosis in a dose-dependent manner.

Twenty-eight days after surgery, HE staining identified histological deviations in brain tissues (Figure 2). The neurons in the control group were arranged in an orderly manner with round cells, pale stained nuclei, clear nuclear membranes, and clear nucleoli. No degeneration, necrosis, or other lesions were observed. In the model group, focal infarction areas were observed in the cortex, hippocampus, and white matter, with large infarction foci and no necrotic material absorption. Histiocyte (foam cell) and nerve cell degeneration were obvious. The number and size of infarcts in the SLT group were significantly lower than those in the model group. Necrosis in the infarcts of the SLT group was not obvious, and necrotic substances were absorbed. The SLT group exhibited no obvious nerve cell degeneration.

### 3.3. Effect of SLT on Memory Impairment Induced by Brain Ischaemia in the MWM Test

**Figure 3:**
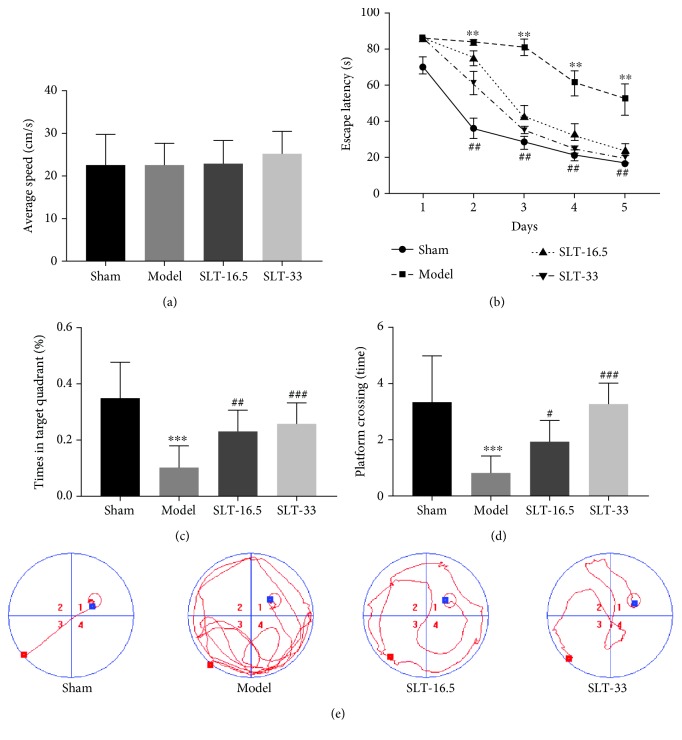
Neuroprotective effects of SLT on the Morris water maze (MWM) test: (a) average swimming speed, (b) escape latency, (c) platform crossing, (d) target quadrant time, and (e) trajectory of swimming. Data are expressed as the mean ± SEM (*n* = 10). ^∗∗^*P* < 0.01 and ^∗∗∗^*P* < 0.001 vs. sham group; ^#^*P* < 0.05, ^##^*P* < 0.01, and ^###^*P* < 0.001 vs. model groups.

The MWM was used to measure changes in learning, memory, and strategy in rats and to determine the effects of SLT. After 5 days of training, all rats found the hidden platform. During this training, the mean swimming speed of the rats in each group was comparable, suggesting that all groups had normal sensory-motor functions and survival motivation (Figure 3(a)). With daily training, the escape latency of the SLT-16.5 group and the SLT-33 group decreased significantly more than that of the model group (*P* < 0.01, model group vs. control group; *P* < 0.01, SLT-33 group vs. model group) (Figure 3(b)). During the retrieval trial, memory retention was assessed by calculating the number of platform crossings and time spent in the target quadrant for each rat. The images of the rat trajectories showed there were fewer platform region crossings in the model group than in the control group (Figure 3(e)). However, the SLT-16.5 group and the SLT-33 group showed significantly greater retention than the model group, measured as more platform crossings (*P* < 0.001, model group vs. control group; *P* < 0.001, SLT-33 group vs. model group) (Figure 3(d)). In addition, we found that with larger doses of SLT, rats spent more time in the target quadrant. The time spent in the target quadrant of the SLT-16.5 group and the SLT-33 group was greater than that of the model group and even reached values observed in the control group (*P* < 0.001, model group vs. control group; *P* < 0.001, SLT-33 group vs. model group) (Figure 3(c)). These results suggest that SLT improved situational memory after ischaemic brain injury.

### 3.4. Effect of SLT on Inflammation Markers

**Figure 4:**
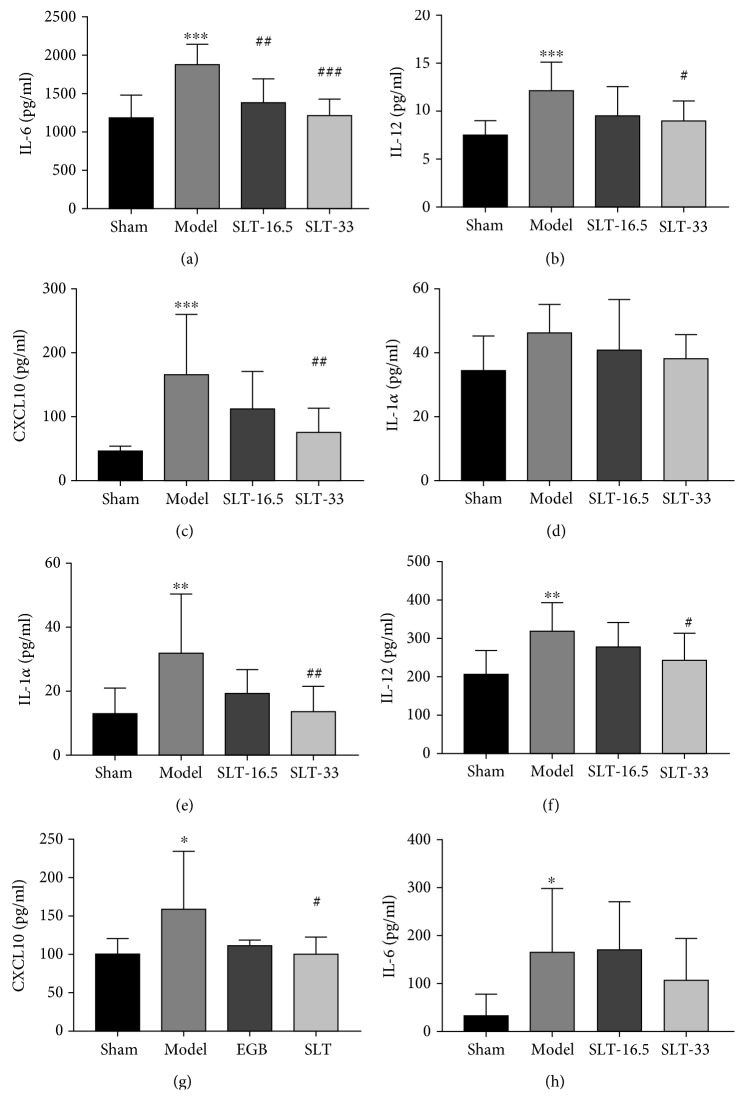
Effects of SLT treatment on the level of cytokines/chemokines in the brain after cerebral ischaemia. (a–f) Analysis showing the relative levels of the proinflammatory mediators IL-1*α*, IL-6, IL-12, and CXCL10 (IP-10) in the brain and in serum; (a–d) was in the brain; (e–h) was in serum. Data are expressed as the mean ± SEM (*n* = 5). ^∗^*P* < 0.05, ^∗∗^*P* < 0.01, and ^∗∗∗^*P* < 0.001 vs. sham group; ^#^*P* < 0.05, ^##^*P* < 0.01, and ^###^*P* < 0.001 vs. the model groups.

There were significant differences in cytokine levels following surgery. After 28 days, the proinflammatory cytokines IL-6, IL-12, and IL-1*α* and chemokine CXCL10 (IP-10) were constant in the control group, while they increased in the ipsilateral hemispheres of the model group, with IL-6, IL-12, and CXCL10 showing significant increases (*P* < 0.001). However, these cytokine levels were significantly and dose-dependently reduced by SLT treatment (*P* < 0.05, SLT-33 group vs. model group; *P* < 0.01, SLT-33 group vs. model group) (Figures 4(a)–4(c)). In addition, the same results were found in the serum. Further analysis showed that the levels of IL-1*α*, IL-12, and CXCL10 in the model group were above those in the control group (*P* < 0.05 for IL-1*α*, IL-12, and CXCL10) (Figures 4(d)–4(f)). Similarly, the cytokine levels were lower in the SLT-33 group than in the ischaemic group (*P* < 0.05, SLT-33 group vs. model group; *P* < 0.01, SLT-33 group vs. model group).

### 3.5. SLT Decreases LCN2 Upregulation in a Rodent Model of Cerebral Ischaemia

**Figure 5:**
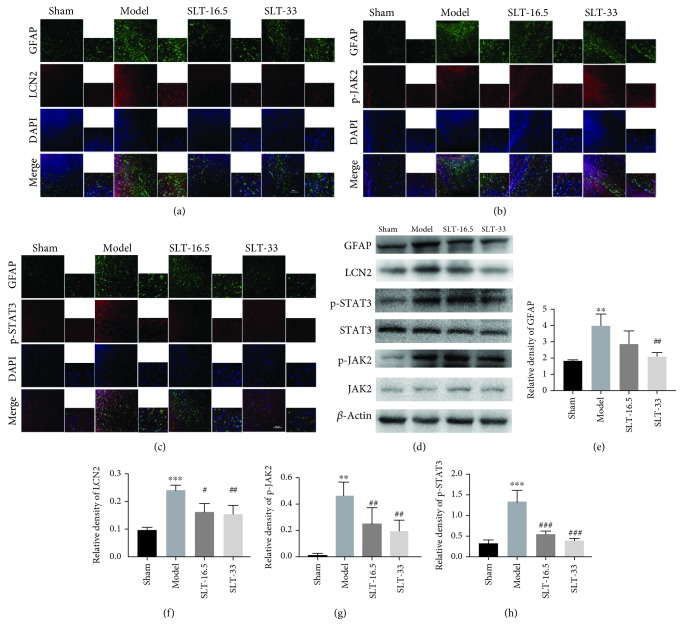
Effects of SLT on the activation of astrocytes and the expression of LCN2, p-JAK2, and p-STAT3, in cerebral ischaemia rats. (a–c) Double immunofluorescence staining for astrocytic LCN2, p-STAT3, p-JAK2, and GFAP expression in the ischaemic penumbra area after cerebral ischaemia. Scale bar = 20 *μ*m. (d–h) Western blots and quantitative analysis of GFAP, LCN2, p-JAK2, and p-STAT3 expression are expressed as the mean ± SEM (*n* = 4). ^∗^*P* < 0.05, ^∗∗^*P* < 0.01, and ^∗∗^*P* < 0.001 vs. sham group; ^#^*P* < 0.05, ^##^*P* < 0.01, and ^###^*P* < 0.001 vs. model groups.

In an *in vivo* study, SLT was demonstrated to prevent cerebral ischaemia-induced neuroinflammation. We further investigated the anti-inflammatory effects using immunofluorescence microscopy and Western blot analysis. Intragastric administration of SLT for 28 days reduced LCN2 secretion due to cerebral ischaemia. The analyses of GFAP and LCN2 were quantified and presented in their relative ratios to *β*-actin expression, and the p-JAK2 and p-STAT3 were quantified in total JAK2 and STAT3 expression. LCN2 and GFAP immunofluorescence staining showed that LCN2 protein expression was induced in GFAP-positive astrocytes, and the rats with cerebral ischaemia had more cortical astrocytes than rats in the control group (Figure 5). Nevertheless, immunostaining showing that SLT (33 mg/kg) repressed astrocytes in the ischaemic hemispheres. The p-STAT3 and p-JAK2 staining in astrocytes was significantly stronger after ischaemia but decreased after SLT treatment (Figures 5(a)–5(c)). The same pattern was observed for GFAP, LCN2, p-STAT3, and p-JAK2 using Western blot analysis (Figures 5(d)–5(h)). Finally, SLT dose-dependently decreased the expression of LCN2, p-STAT3, and p-JAK2 in rats exposed to ischaemia (*P* < 0.01).

### 3.6. SLT Decreases OGD-Induced Injury in Astrocytes

A CCK8 assay showed that OGD resulted in a significant reduction in cell viability. SLT (3.125~100 mg/l) displayed a dose-dependent toxicity to human astrocytes (CCK8 assay), yet the lower concentrations of SLT (2.5~50 mg/l) reduced the damage caused by OGD. Because the protective effect became apparent at 2.5 mg/l (*P* < 0.05, vs. OGD group), no higher concentrations were used in the *in vitro* tests ([Supplementary-material supplementary-material-1]).

**Figure 6:**
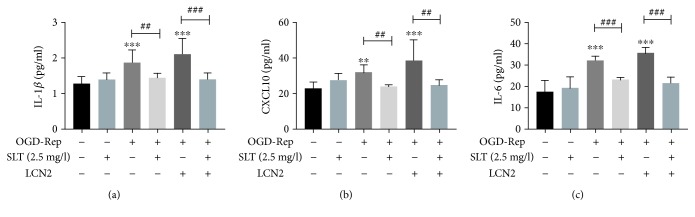
SLT suppressed OGD-induced inflammation in astrocytes in vitro. (a–c) Analysis showing the relative levels of the proinflammatory mediators IL-1*β*, IL-6, and CXCL10 (IP-10). Data are expressed as the mean ± SEM (*n* = 5). ^∗^*P* < 0.05, ^∗∗^*P* < 0.01, and ^∗∗∗^*P* < 0.001 vs. control group; ^#^*P* < 0.05, ^##^*P* < 0.01, and ^###^*P* < 0.001 vs. the indicated groups.

To further investigate the specific effects of LCN2, human astrocytes were subjected to the expression vector LCN2 by lentiviruses 24 hours before OGD induction. The cells were induced in an OGD environment to imitate ischaemia, and SLT treatment markedly decreased the levels of the proinflammatory cytokines IL-6, IL-1*β*, and CXCL10 (*P* < 0.05). LCN2 overexpression enhanced this difference (*P* < 0.05, vs. control group) (Figures 6(a)–6(c)).

**Figure 7:**
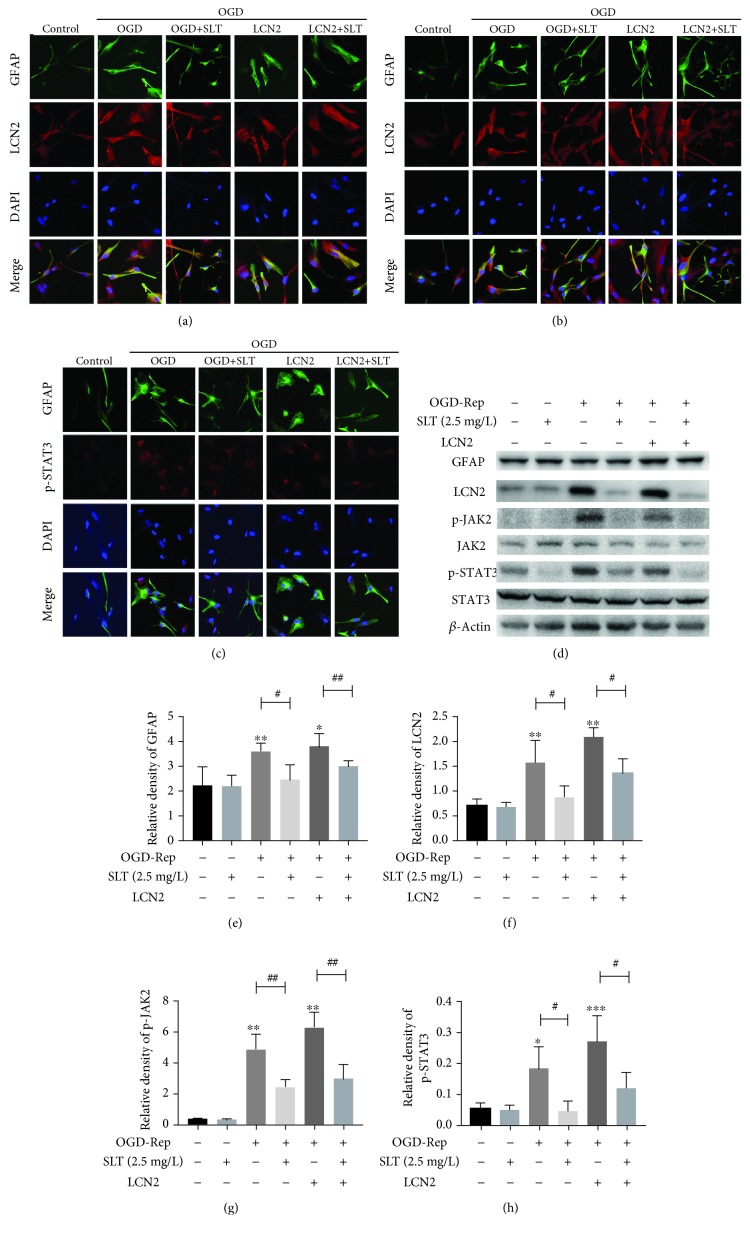
The effects of SLT on the activation of astrocytes and the expression of astrocytic LCN2, p-JAK2, and p-STAT3 after OGD induction in vitro. (a–h) Double immunofluorescence staining for GFAP, LCN2, p-JAK2, and p-STAT3 in astrocytes after OGD induction. Scale bar = 20 *μ*m. Western blots and quantitative analysis of GFAP, LCN2, p-JAK2, and p-STAT3 expression are expressed as the mean ± SEM (*n* = 3). ^∗^*P* < 0.05 and ^∗∗^*P* < 0.01 vs. control group; ^#^*P* < 0.05 and ^##^*P* < 0.01 vs. the indicated groups.

The morphology of astrocytes was observed by GFAP staining (Figures 7(a)–7(c)). Prolonged cellular protuberances confirmed OGD-induced astrocyte activation, and LCN2 overexpression enhanced this effect. The OGD group showed higher levels of GFAP, LCN2, p-JAK2, and p-STAT3 than the control group (*P* < 0.05, *P* < 0.01) (Figures 7(a)–7(h)), and overexpression of LCN2 enhanced these effects (Figure 7). However, SLT negated these increases after OGD induction (*P* < 0.05, *P* < 0.01).

## 4. Discussion

High levels of LCN2 trigger inflammation and later reduce cognitive activity, making LCN2 a possible target in anti-inflammation therapies [29, 30]. We have confirmed the efficacy of SLT in regulating reactive astrocytes and LCN2 expression in a cerebral ischaemic model. We found that SLT can protect cognitive function, significantly reducing stroke injury and protecting the cerebral cortex from injury by reducing cerebral infarction volume in ischaemic rats. Furthermore, SLT improves astrocyte activation, reduces STAT3 and JAK2 phosphorylation, and reduces the expression of LCN2 *in vitro* and *in vivo*. These results suggest that SLT plays a powerful therapeutic role in cerebral ischaemia by reducing astrocytic LCN2 release and inhibiting neuroinflammatory injury through the JAK2/STAT3 pathway.

The neuronal inflammation has emerged as a crucial element in stroke, both in the beginning and later stages. Recent reviews have noted that lymphocytes and inflammatory mediators promulgate the development of neurological lesions and deficits, although most of these findings have been from experimental stroke models [31–34]. Other studies have shown that inflammation may also play a role in the aetiology of mild cognitive impairment (MCI) [35, 36]. As mentioned in Introduction, assorted traditional Chinese medicine formulations for the treatment of stroke, such as SLT, have been developed [15]. SLT is composed of ginseng extract (ginseng total saponins), *G. biloba* extract (*G. biloba* total flavonoids), and saffron extract (saffron total glycosides). Pharmacodynamic reports have shown that SLT meaningfully improves problems produced by cerebral ischaemia as well as learning and memory ability in experimental models of cerebral ischaemia. Neurocognitive and cardiovascular functions improved in normal adults after a one-week regime on SLT [15]. Additionally, from 2012 to 2014, an international multicentre phase II clinical trial of SLT use in patients with mild to moderate VaD showed that SLT improved cognition and daily functioning in Chinese patients [25]. SLT reportedly prevents H_2_O_2_-induced endothelial cell damage via a direct decrease in intracellular reactive oxygen species (ROS) generation and increase in superoxide dismutase (SOD) activity [37]. In our present study, SLT improved cognitive function of cerebral ischaemic model rats in the MWM test. Neurological deficits were also attenuated in cerebral ischaemic rats treated with SLT. Hence, these results provide the first evidence that SLT alleviates memory impairment in animal models.

Proinflammatory cytokines and chemokines are small (8–12 kDa) proteins with numerous purposes. In the CNS, proinflammatory cytokines and chemokines facilitate innate and adaptive immune responses, indicating the need for leukocyte recruitment as well as astrocyte activation during neuroinflammation [38, 39]. Although cytokines/chemokines function by starting the inflammatory response and the recruitment of peripheral immune cells to help remove harmful stimulation, chemokines are involved in certain neurological diseases involving inflammation of nerves, including neurodegenerative dementia, Alzheimer's disease, multiple sclerosis, traumatic brain injury, and certain types of meningitis [40, 41]. In the current study, SLT notably reduced the level of proinflammatory cytokines and chemokines, including IL-6, IL-12, and CXCL10, in tissues adjacent to the damage and in the serum. Additionally, SLT reduced these proinflammatory cytokines and chemokines in the astrocytic supernatant.

Astrogliosis can cause extension of cell processes and swelling of cell bodies after injury. Reactive gliosis has been shown to be sustained for up to 60 days after controlled cortical impact injury in rats, signifying a continuing response of astrocytes to brain injury [42]. However, the lack of effective treatment for astrogliosis has become a focus of clinical treatment. LCN2, a secreted glycoprotein in the lipocalin superfamily, has been reported to play a harmful role in ischaemic brains and neurodegenerative diseases [14, 43–45]. Neuroinflammation due to brain injury or neurodegenerative diseases initiates the secretion of LCN2 from astrocytes, microglia, endothelial cells, and neurons. Inflammatory mediators released by activated astrocytes play an important role in cell migration and the recruitment of glial cells to the injury site. Our data suggest that astrocytes were significantly activated after cerebral ischaemia and that LCN2 was upregulated throughout the cerebral ischaemic cortex. SLT treatment significantly inhibited astrocyte activation and decreased LCN2 expression. The same phenomenon was also found in OGD-induced astrocytes.

Over the years, research into the cellular and molecular pathways related to astrogliosis has provided a basis for future treatment options for memory disorders. LCN2 plays a critical role in astrocytic reactions [46]. JAK2-STAT3 activation is implicated in haematopoiesis [47], immune responses [48], morphogenesis [49], gliogenesis [50], and the regulation of memory formation. Previous studies have demonstrated that JAK2/STAT3 is essential for the induction of LCN2 [51]. Specifically, upregulation of CXCL10 by the JAK2/STAT3 pathway in astrocytes plays a central role in LCN2-induced cell migration. Thus, the neuroinflammatory pathway could be regulated by inhibiting the constitutive secretion of LCN2. In these experiments, astrocytic p-STAT3 and p-JAK2 were highly activated in the ischaemic hemisphere after cerebral ischaemia *in vivo* and after OGD *in vitro*. However, SLT inhibited JAK2-STAT3 activation in the cerebral ischaemia model and OGD-related neuroinflammatory alterations. Meanwhile, p-STAT3 and p-JAK2 were upregulated by LCN2 overexpression *in vitro*, but SLT inhibited this effect. These studies indicate that the LCN2 partially mediates JAK2/STAT3 pathway upregulation of GFAP expression in astrocytes [51]. Thus, inactivation of LCN2 by SLT confers significant antineuroinflammatory and antiastrogliotic roles in the brain. These results do not explain all the mechanisms of SLT protection, and although the reduction in LCN2 expression is considered to be a key mechanism, SLT, as a multicomponent drug, may have other indirect neuroprotective effects. Additionally, although most of the data from experiments indicated that the effects of LCN2 were largely mediated through JAK2-STAT3 inhibition, we cannot rule out the possibility of some nonspecific effects of LCN2.

## 5. Conclusion

Overall, this study indicates that SLT has antiastrogliosis and antineuroinflammatory effects and improves neuronal survival and memory deficiency in a cerebral ischaemia model and OGD-induced astrocytes. As a standardized herb formula, SLT has a potential application for neuroinflammatory diseases, such as VaD, through the inactivation of LCN2.

## Data Availability

All the datasets and materials supporting the conclusions of this article are provided in the manuscript, which include the article and the additional files. Some pictures of Figure 7 were reproduced from Zhang et al. [20] (under the Creative Commons Attribution License/public domain).

## Abbreviations

CCK8: Cell counting kit-8
CNS: Central nervous system
CXCL: Chemokine (C-X-C motif) ligand
DALYs: Disability-adjusted life years
DAPI: 4′,6-Diamidino-2-phenylindole
FBS: Fetal bovine serum
GFAP: Glial fibrillary acidic protein
HE: Hematoxylin-eosin
HPLC: High-performance liquid chromatography
IL-1*α*: Interleukin-1*α*
IL-1*β*: Interleukin-1*β*
IL-6: Interleukin-6
JAK2: Janus kinase 2
LCN2: Lipocalin-2
OGD: Oxygen-glucose deprivation
POAD: Peripheral occlusive arterial disease
SLT: Sailuotong capsule
STAT3: Signal transducer and activator of transcription 3
TNF-*α*: Tumor necrosis factor-alpha
TTC: Triphenyltetrazolium chloride
UV: Ultraviolet
VaD: Vascular dementia.

## Supplementary Materials

Supplementary Materials Figure S1: the effect of SLT on cell viability in astrocytes. (A) MTT assay detecting the cell viability treated with different dosages of SLT for 24 h. (B) MTT assay detecting the cell viability treated with different dosages of SLT for 6 h during OGD induction. Data are expressed as the mean ± SEM (*n* = 6). ^∗^*P* < 0.05, ^∗∗^*P* < 0.01, and ^∗∗^*P* < 0.001 vs. the control groups; ^#^*P* < 0.05, ^##^*P* < 0.05, and ^###^*P* < 0.001 vs. the OGD groups. Figure S2: analysis showing the relative levels of the proinflammatory mediators IL-1*β*, IL-6, and CXCL10 (IP-10) in the control cell and LCN2 overexpression cell conditioned in medium without OGD deprivation. Figure S3: the expression of astrocytic LCN2 in vitro without OGD deprivation.

## References

[1] Hankey G. J. (2017). Stroke. *The Lancet*.

[2] Ivan C. S., Seshadri S., Beiser A. (2004). Dementia after stroke: the Framingham Study. *Stroke*.

[3] Anrather J., Iadecola C. (2016). Inflammation and stroke: an overview. *Neurotherapeutics*.

[4] Petrovic-Djergovic D., Goonewardena S. N., Pinsky D. J. (2016). Inflammatory disequilibrium in stroke. *Circulation Research*.

[5] Pekny M., Wilhelmsson U., Bogestål Y. R., Pekna M. (2007). The role of astrocytes and complement system in neural plasticity. *International Review of Neurobiology*.

[6] McKimmie C. S., Graham G. J. (2010). Astrocytes modulate the chemokine network in a pathogen-specific manner. *Biochemical and Biophysical Research Communications*.

[7] Li H., Zhang N., Lin H. Y. (2014). Histological, cellular and behavioral assessments of stroke outcomes after photothrombosis-induced ischemia in adult mice. *BMC Neuroscience*.

[8] Kehrer J. P. (2010). Lipocalin-2: pro- or anti-apoptotic. *Cell Biology and Toxicology*.

[9] Yang J., Bielenberg D. R., Rodig S. J. (2009). Lipocalin 2 promotes breast cancer progression. *Proceedings of the National Academy of Sciences of the United States of America*.

[10] Biessels G. J., Reagan L. P. (2015). Hippocampal insulin resistance and cognitive dysfunction. *Nature Reviews Neuroscience*.

[11] Wu Z. L., Ciallella J. R., Flood D. G., O’Kane T. M., Bozyczko-Coyne D., Savage M. J. (2006). Comparative analysis of cortical gene expression in mouse models of Alzheimer’s disease. *Neurobiology of Aging*.

[12] Marques F., Rodrigues A. J., Sousa J. C. (2008). Lipocalin 2 is a choroid plexus acute-phase protein. *Journal of Cerebral Blood Flow & Metabolism*.

[13] Eikelenboom P., Veerhuis R., Scheper W., Rozemuller A. J. M., van Gool W. A., Hoozemans J. J. M. (2006). The significance of neuroinflammation in understanding Alzheimer’s disease. *Journal of Neural Transmission*.

[14] Jin M., Kim J. H., Jang E. (2014). Lipocalin-2 deficiency attenuates neuroinflammation and brain injury after transient middle cerebral artery occlusion in mice. *Journal of Cerebral Blood Flow & Metabolism*.

[15] Steiner G. Z., Yeung A., Liu J. X. (2015). The effect of Sailuotong (SLT) on neurocognitive and cardiovascular function in healthy adults: a randomised, double-blind, placebo controlled crossover pilot trial. *BMC Complementary and Alternative Medicine*.

[16] Bridi R., Crossetti F. P., Steffen V. M., Henriques A. T. (2001). The antioxidant activity of standardized extract of *Ginkgo biloba* (EGb 761) in rats. *Phytotherapy Research*.

[17] Lee E. J., Chen H. Y., Wu T. S., Chen T. Y., Ayoub I. A., Maynard K. I. (2002). Acute administration of *Ginkgo biloba* extract (EGb 761) affords neuroprotection against permanent and transient focal cerebral ischemia in Sprague-Dawley rats. *Journal of Neuroscience Research*.

[18] Domoráková I., Burda J., Mechírová E., Feriková M. (2006). Mapping of rat hippocampal neurons with NeuN after ischemia/reperfusion and Ginkgo biloba extract (EGb 761) pretreatment. *Cellular and Molecular Neurobiology*.

[19] Paganelli R. A., Benetoli A., Milani H. (2006). Sustained neuroprotection and facilitation of behavioral recovery by the *Ginkgo biloba* extract, EGb 761, after transient forebrain ischemia in rats. *Behavioural Brain Research*.

[20] Zhang Y., Liu J., Yang B. (2018). *Ginkgo biloba* extract inhibits astrocytic lipocalin-2 expression and alleviates neuroinflammatory injury via the JAK2/STAT3 pathway after ischemic brain stroke. *Frontiers in Pharmacology*.

[21] Lee T. F., Shiao Y. J., Chen C. F., Wang L. C. H. (2001). Effect of ginseng saponins on *β*-amyloid-suppressed acetylcholine release from rat hippocampal slices. *Planta Medica*.

[22] Shen L., Zhang J. (2007). NMDA receptor and iNOS are involved in the effects of ginsenoside Rg1 on hippocampal neurogenesis in ischemic gerbils. *Neurological Research*.

[23] Zhang G., Liu A., Zhou Y., San X., Jin T., Jin Y. (2008). Panax ginseng ginsenoside-Rg_2_ protects memory impairment via anti-apoptosis in a rat model with vascular dementia. *Journal of Ethnopharmacology*.

[24] Zhao H., Li Q., Pei X. (2009). Long-term ginsenoside administration prevents memory impairment in aged C57BL/6J mice by up-regulating the synaptic plasticity-related proteins in hippocampus. *Behavioural Brain Research*.

[25] Jia J., Wei C., Chen S. (2018). Efficacy and safety of the compound Chinese medicine SaiLuoTong in vascular dementia: a randomized clinical trial. *Alzheimer's & Dementia: Translational Research & Clinical Interventions*.

[26] Zhang Y., Miao L., Lin L., Ren C. Y., Liu J. X., Cui Y. M. (2018). Repeated administration of Sailuotong, a fixed combination of *Panax ginseng, Ginkgo biloba, and Crocus sativus* extracts for vascular dementia, alters CYP450 activities in rats. *Phytomedicine*.

[27] Bederson J. B., Pitts L. H., Tsuji M., Nishimura M. C., Davis R. L., Bartkowski H. (1986). Rat middle cerebral artery occlusion: evaluation of the model and development of a neurologic examination. *Stroke*.

[28] Morris R. (1984). Developments of a water-maze procedure for studying spatial learning in the rat. *Journal of Neuroscience Methods*.

[29] Choi J., Lee H. W., Suk K. (2011). Increased plasma levels of lipocalin 2 in mild cognitive impairment. *Journal of the Neurological Sciences*.

[30] Jang E., Lee S., Kim J. H. (2013). Secreted protein lipocalin-2 promotes microglial M1 polarization. *The FASEB Journal*.

[31] Wang J. (2010). Preclinical and clinical research on inflammation after intracerebral hemorrhage. *Progress in Neurobiology*.

[32] Iadecola C., Anrather J. (2011). The immunology of stroke: from mechanisms to translation. *Nature Medicine*.

[33] Macrez R., Ali C., Toutirais O. (2011). Stroke and the immune system: from pathophysiology to new therapeutic strategies. *The Lancet Neurology*.

[34] Chamorro Á., Meisel A., Planas A. M., Urra X., van de Beek D., Veltkamp R. (2012). The immunology of acute stroke. *Nature Reviews Neurology*.

[35] Yaffe K., Kanaya A., Lindquist K. (2004). The metabolic syndrome, inflammation, and risk of cognitive decline. *JAMA*.

[36] Arfanakis K., Fleischman D. A., Grisot G. (2013). Systemic inflammation in non-demented elderly human subjects: brain microstructure and cognition. *PloS One*.

[37] Seto S. W., Chang D., Ko W. (2017). Sailuotong prevents hydrogen peroxide (H_2_O_2_)-induced injury in EA.hy926 cells. *International Journal of Molecular Sciences*.

[38] Mennicken F., Maki R., de Souza E. B., Quirion R. (1999). Chemokines and chemokine receptors in the CNS: a possible role in neuroinflammation and patterning. *Trends in Pharmacological Sciences*.

[39] Allan S. M., Rothwell N. J. (2001). Cytokines and acute neurodegeneration. *Nature Reviews Neuroscience*.

[40] Mastroianni C. M., Lancella L., Mengoni F. (1998). Chemokine profiles in the cerebrospinal fluid (CSF) during the course of pyogenic and tuberculous meningitis. *Clinical and Experimental Immunology*.

[41] Gyoneva S., Ransohoff R. M. (2015). Inflammatory reaction after traumatic brain injury: therapeutic potential of targeting cell-cell communication by chemokines. *Trends in Pharmacological Sciences*.

[42] Sosunov A. A., Wu X., Tsankova N. M., Guilfoyle E., McKhann G. M., Goldman J. E. (2014). Phenotypic heterogeneity and plasticity of isocortical and hippocampal astrocytes in the human brain. *Journal of Neuroscience*.

[43] Naudé P. J. W., Nyakas C., Eiden L. E. (2012). Lipocalin 2: novel component of proinflammatory signaling in Alzheimer’s disease. *The FASEB Journal*.

[44] Bi F., Huang C., Tong J. (2013). Reactive astrocytes secrete lcn2 to promote neuron death. *Proceedings of the National Academy of Sciences of the United States of America*.

[45] Suk K. (2016). Lipocalin-2 as a therapeutic target for brain injury: an astrocentric perspective. *Progress in Neurobiology*.

[46] Lee S., Park J. Y., Lee W. H. (2009). Lipocalin-2 is an autocrine mediator of reactive astrocytosis. *Journal of Neuroscience*.

[47] Kisseleva T., Bhattacharya S., Braunstein J., Schindler C. W. (2002). Signaling through the JAK/STAT pathway, recent advances and future challenges. *Gene*.

[48] Ivashkiv L. B. (2000). Jak-STAT signaling pathways in cells of the immune system. *Reviews in Immunogenetics*.

[49] Schindler C. W. (2002). Series introduction: JAK-STAT signaling in human disease. *Journal of Clinical Investigation*.

[50] Na Y. J., Jin J. K., Kim J. I., Choi E. K., Carp R. I., Kim Y. S. (2007). JAK-STAT signaling pathway mediates astrogliosis in brains of scrapie-infected mice. *Journal of Neurochemistry*.

[51] Lee S., Kim J. H., Kim J. H. (2011). Lipocalin-2 is a chemokine inducer in the central nervous system: role of chemokine ligand 10 (CXCL10) in lipocalin-2-induced cell migration. *Journal of Biological Chemistry*.

