# Supplementary material for: Sailuotong Capsule Prevents the Cerebral Ischaemia-Induced Neuroinflammation and Impairment of Recognition Memory through Inhibition of LCN2 Expression
Source: Oxid Med Cell Longev. 2019 Sep 3;2019:8416105. doi: 10.1155/2019/8416105 (PMC6745154; doi:10.1155/2019/8416105)
Supplement: Supplementary Materials — Figure S1: the effect of SLT on cell viability in astrocytes. (A) MTT assay detecting the cell viability treated with different dosages of SLT for 24 h. (B) MTT assay detecting the cell viability treated with different dosages of SLT for 6 h during OGD induction. Data are expressed as the mean ± SEM (n = 6). ∗P < 0.05, ∗∗P < 0.01, and ∗∗P < 0.001 vs. the control groups; #P < 0.05, ##P < 0.05, and ###P < 0.001 vs. the OGD groups. Figure S2: analysis showing the relative levels of the proinflammatory mediators IL-1β, IL-6, and CXCL10 (IP-10) in the control cell and LCN2 overexpression cell conditioned in medium without OGD deprivation. Figure S3: the expression of astrocytic LCN2 in vitro without OGD deprivation. [file 8416105.f1.pdf]

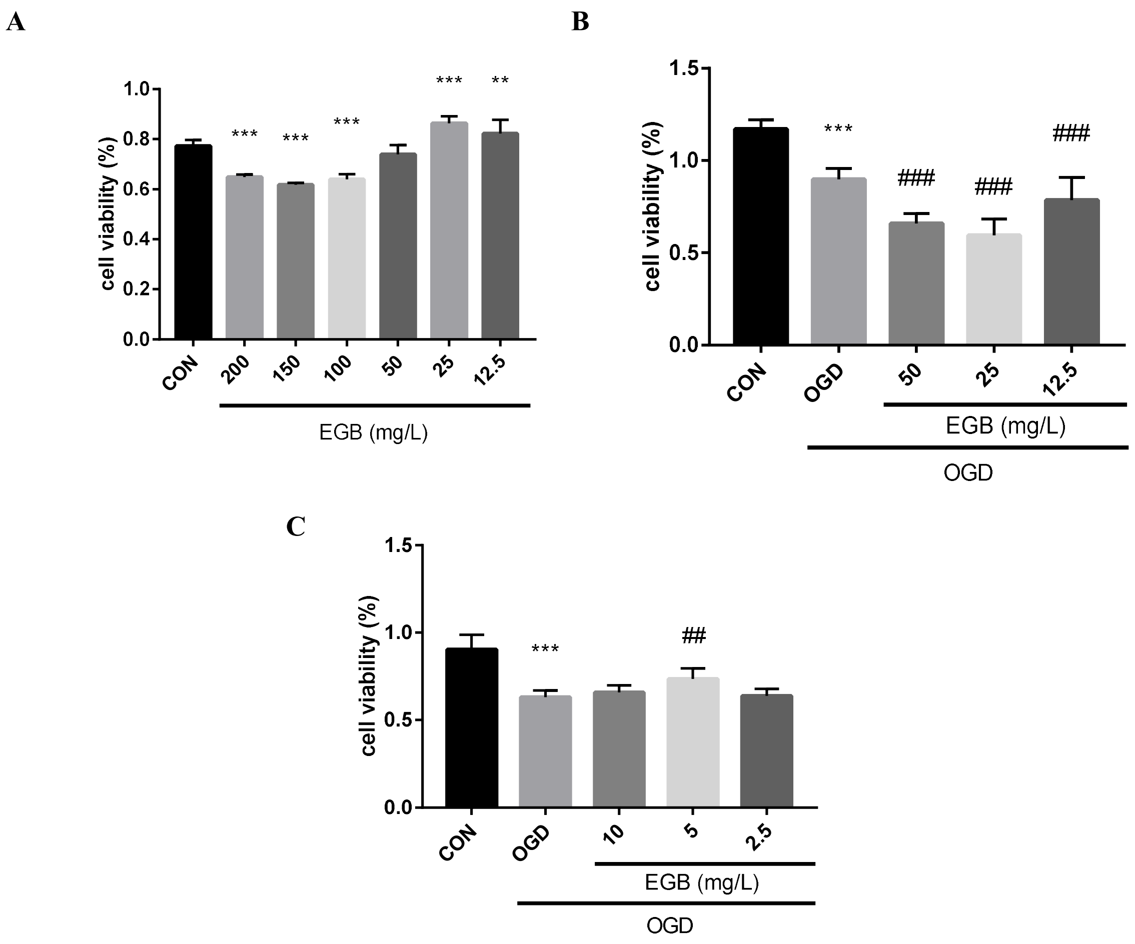

Figure S1. The effect of EGB on cell viability in astrocytes.

A. MTT assay detecting the cell viability treated with different dosages of EGB for 24 h.

B-C MTT assay detecting the cell viability treated with different dosages of EGB for 6h during OGD induction. Data are expressed as mean ± SEM (n = 6). *P < 0.05, **P < 0.01, ** P < 0.001 *vs.* the control groups, $^{\#}P$ < 0.05, $^{\#\#}P$ < 0.05 and $^{\#\#\#}P$ < 0.001 *vs.* the OGD groups.

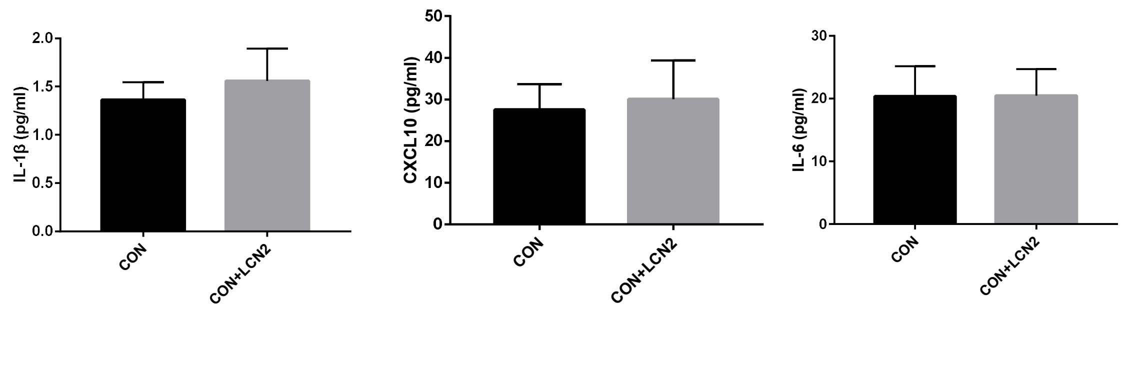

Figure S2 Analysis showing the relative levels of the pro-inflammatory mediators IL-1β, IL-6, and CXCL10 (IP-10) in control cell and LCN2 overexpression cell conditioned in medium without OGD deprivation.

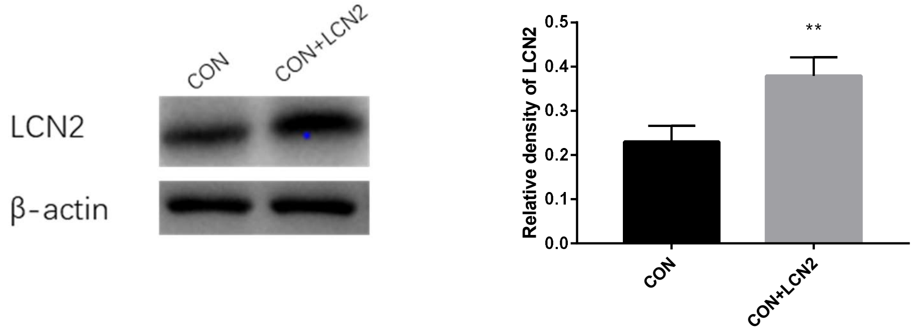

Figure S3 The expression of astrocytic LCN2 in vitro without OGD deprivation.
